# Automatic extraction of informal topics from online suicidal ideation

**DOI:** 10.1186/s12859-018-2197-z

**Published:** 2018-06-13

**Authors:** Reilly N. Grant, David Kucher, Ana M. León, Jonathan F. Gemmell, Daniela S. Raicu, Samah J. Fodeh

**Affiliations:** 1 Grinnell College, Grinnell, IA USA; 2 University of Michigan, Ann Arbor, MI USA; 3 University of Juárez Autónoma de Tabasco, Villahermosa, Tab., 86040 Mexico; 4 School of Computing, DePaul University, Chicago, IL USA; 5 Yale Center for Medical Informatics, Yale University, New Haven, CT USA

**Keywords:** Suicidal ideation, Word2Vec, Text mining

## Abstract

**Background:**

Suicide is an alarming public health problem accounting for a considerable number of deaths each year worldwide. Many more individuals contemplate suicide. Understanding the attributes, characteristics, and exposures correlated with suicide remains an urgent and significant problem. As social networking sites have become more common, users have adopted these sites to talk about intensely personal topics, among them their thoughts about suicide. Such data has previously been evaluated by analyzing the language features of social media posts and using factors derived by domain experts to identify at-risk users.

**Results:**

In this work, we automatically extract informal latent recurring topics of suicidal ideation found in social media posts. Our evaluation demonstrates that we are able to automatically reproduce many of the expertly determined risk factors for suicide. Moreover, we identify many informal latent topics related to suicide ideation such as concerns over health, work, self-image, and financial issues.

**Conclusions:**

These informal topics topics can be more specific or more general. Some of our topics express meaningful ideas not contained in the risk factors and some risk factors do not have complimentary latent topics. In short, our analysis of the latent topics extracted from social media containing suicidal ideations suggests that users of these systems express ideas that are complementary to the topics defined by experts but differ in their scope, focus, and precision of language.

## Background

Suicide, the act of causing one’s own death, is the tenth leading cause of mortality in the United States and is estimated to cost 44.6 billion dollars per year. This understates the severity of the problem, as for every attempted suicide, there are nearly 10 times as many people who contemplate suicide [1]. Suicidal ideation includes a wide range of thoughts from momentary consideration to extensive planning or incomplete attempts. The scope and impact of this mental health issue make understanding it a public health priority.

When discussing their ideations, many individuals often reference common symptoms: feeling helpless, feeling alone, excessive fatigue, low self-esteem, the feeling that one’s mind is racing, or excessive focus on dormant goals [2]. Understanding the common themes in suicidal ideation can help us understand the patterns behind suicidal thoughts, ultimately leading to treatment and prevention.

Clinical research toward understanding suicide has identified several risk factors. Mental disorders such as depression, schizophrenia, alcoholism, and substance abuse all play a contributing role. Additionally, the emotional stress caused by bullying, interpersonal relationships, and finances are also important factors [3]. However, these descriptions of suicidal ideation often capture a clinical viewpoint.

With the rise in sophistication and acceptance of online social networks, individuals contemplating suicide have increasingly expressed their suicidal ideation in online forums, tweets, and other online media. The result is a vast collaborative description of the thoughts and motivations associated with suicide. In this paper, we leverage advanced topic modeling techniques to extract informal latent topics from this data.

Topic modeling is a machine learning approach for eliciting abstract topics from a collection of documents. This approach can be leveraged to discover common themes present in online posts such as depression, drug use, or violence. The idea of “depression” might be captured by a collection of related words such as “pain”, “feelings”, “fear”, “stress”, and “suffering”.

In this paper, we perform topic modeling on over 130,000 submissions to r/SuicideWatch, an online forum described as a place of support for those suffering suicidal thoughts. We begin by learning semantic embeddings for words in the posts via a shallow, two-layer neural network. Then we cluster the words into topics producing informally generated latent topics. Finally, we evaluate these informal topics by comparing them to suicidal risk factors and common themes identified by mental health professionals [3].

Our experimental results reveal that we are able to automatically generate quality embeddings for words and corresponding topic models. Many of these topic models correspond to risk factors that domain experts have previously proposed. In some cases, our topic models were more specific, focusing on a narrow interpretation of the risk factor. In other cases, our topics were more broad, encompassing multiple risk factors at once. This suggests that the topics extracted from social media posts created by those experiencing suicidal ideation may have a different focus and specificity than those generated by mental health professionals.

## Related work

Researchers have previously attempted to use the massive amount of data generated through social media to characterize the mental health of users [4, 5], leading to the development of computational tools [6]. Attempts have been made to predict depression, identify suicidal Twitter posts, and analyze the effect of suicide in the media on suicidal ideation in social platforms [7–9]. Risk factors of suicide [10, 11] identified by domain experts are often leveraged in such studies.

A common tool used to analyze social media posts is the Linguistic Inquiry and Word Count (LIWC) [6]. Progress has been made using this tool to analyze text related to suicide and depression, often in social media posts [4, 7, 8, 12–14].

An early study used the LIWC on Twitter to analyze the impact of depression on social media activity [7]. Twitter data has been used to analyze suicidal ideation [5, 9, 15]. In one study, tweets were filtered by using specific search terms which were associated with 12 suicide risk factors [5]. The twelve risk factors include bullying, depressive feelings, depression symptoms, drug abuse, family violence/discord, gun ownership, impulsively, prior suicide attempts, psychological disorders, self-harm, suicide around the individual, and suicide ideation [10, 11]. We also evaluate the twelve risk factors identified in these studies. These researchers found that the volume of suicide-related tweets correlated to suicide rates by U.S. state, showing that Twitter data could be indicative of a population’s mental health.

One study used human “coders” to label tweets according to their level of concern with respect to suicide. Language models were then used to predict the appropriate concern for new tweets [9]. Another study analyzed the content of Twitter users prior to their public declaration of a suicide attempt and found that there may be indications of suicidal ideation based on posts leading up to a suicide attempt [15].

There have also been studies which focused on the social media platform Reddit, specifically the subreddit called r/SuicideWatch. One study analyzed changes in suicide content in the wake of celebrity suicides by measuring post volume and modeling topics in the text [8]. Another study observed the propensity of users discussing mental health issues to transition into discussing suicidal [13]. The language that people use in Reddit has been shown to differ between subreddits focused on different mental health concerns [14].

In this work, we leverage computationally generated language models to explore suicidal ideation. Examples of language models include simple bag-of-word models [16] and extend to more robust models such as probabilistic latent semantic analysis [17], latent dirichlet allocation [18], and Word2Vec [19, 20]. Such language models have been used to explore numerous topics such as comparing topics in data [21], recommendation systems [22], and different languages [23]. We focus on the Word2Vec language model developed by Mikolov et al. [19, 20].

Our work extends upon these previous efforts in the following ways. Rather than using pre-defined risk factors or labeled data to identify at-risk users, we automatically discover topics from the users’ posts by leveraging Word2Vec language models. We compare the latent topics identified in posts to risk factors proposed by domain experts.

## Methods

**Fig. 1:**
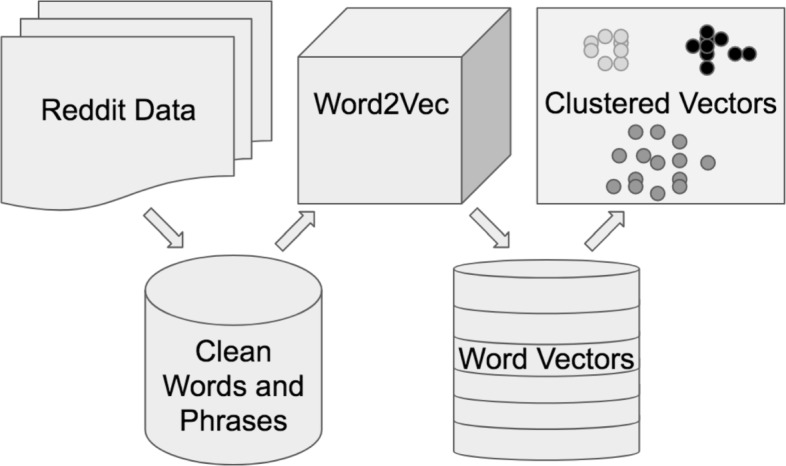
Flowchart of methodology. This describes our process of converting r/SuicideWatch data to a language model, then clustering the vectors provided by Word2Vec to identify the informal latent topics present in the posts

In this section, we provide a detailed description of our procedure including how we represent words with Word2Vec models and then use *k*-means clustering to produce topics in text data. See Fig. 1.

### Word embeddings

We represent words as a vector of real numbers [24]. More formally, each word $\vec {w}$ is represented as: $\vec {w} = \langle \phi (i_{1}), \phi (i_{2})...\phi (i_{n}) \rangle $ where *ϕ*(*i*_1_) through *ϕ*(*i*_*n*_) represent the weight of the *i*th word in the vector space.

We can think of these word representations as populating a high dimensional space where the relative locations contain semantic information. For example, in previous work, the relationship of a country to its capital city has been represented by their relative position in the vector space [19, 20]. There are several methods for learning these weights; we leverage Word2Vec.

### Word2Vec

Many topic modeling algorithms exist, including latent semantic indexing, latent Dirichlet allocation, and non-negative matrix factorization. In this work, we turn our attention to Word2Vec, which has been argued to have many advantages over these earlier algorithms [19, 20].

**Fig. 2:**
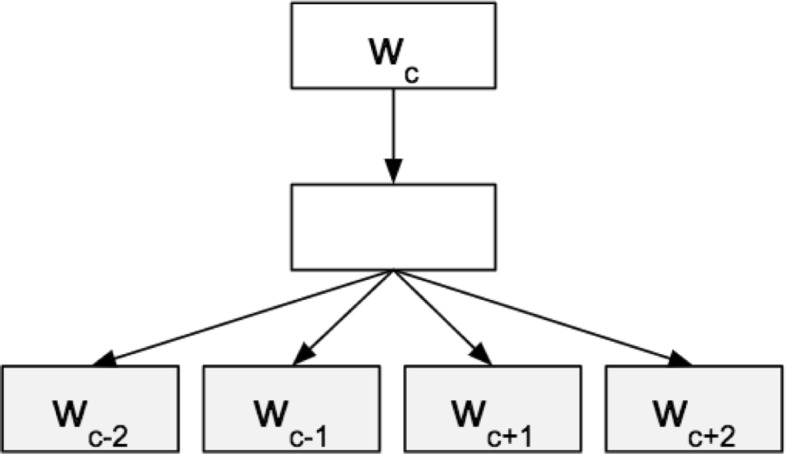
Architecture for the skip-gram model. The skip-gram model predicts the distributed representations of neighbors given a word. In this figure, the representation has a window size of 2, where *w*_*c*_ is the target word being evaluated, and *w*_*c*+*i*_ denotes the surrounding context words

Word2Vec describes two implementations of a shallow neural network, the continuous bag of words (CBOW) model and the skip-gram model. We focus on the skip-gram model in this work, which learns vector representations of words by predicting neighboring words in a text. See Fig. 2.

Common words such as “the” add little meaning to the model and add computational time. Instead of using these words, the model often skips over them and goes to the next word when training. Word2Vec does this by using sub-sampling, a probabilistic approach with the most common words having the greatest chance of being ignored, and the least common words having the least chance of being ignored.

In contrast to many other neural network models, the skip-gram model includes only a single hidden layer, dramatically reducing both training time and complexity [19, 20]. Learning the word representation is achieved by performing back-propagation on our training examples. Instead of updating each of the many neurons used in the neural network, negative sampling [20] updates a small, specified amount of neurons. Since one of the most computationally expensive parts of training a neural network is the act of updating the weights, this technique greatly reduces the training time. Finally, the softmax function normalizes the output of the neural network, so that sum of all outputs is equal to 1.

Word2Vec capitalizes on the fact that similar words should have similar probabilities of appearing in the same context. Therefore the vector representations of similar words are “close” in vector space, often capturing rich semantic characteristics. It has been previously shown that Word2Vec performs accurately on tasks involving word similarity, analogy discovery, and text completion [20].

### Clustering

The word representations are useful in their own right, often containing rich semantic information. However, using these representations as input into other algorithms, such as clustering, can produce meaningful collections of related words. Clustering is a technique wherein items are grouped together based on their similarity. Items in a cluster are “near” one another and “distant” from items in other clusters. In this work, we rely on Euclidean distance because we are interested in the relative positions of the representations in the vector space.

We leverage the *k*-means clustering algorithm [25] to produce clusters of words. We choose *k*-means clustering due to its simplicity and ability to create localized, spherical clusters. *K*-means begins with cluster centers at *k* random locations in the vector space. The algorithm assigns every item, in this case words to the nearest cluster. For each cluster, the mean of all items is calculated, and the cluster center is moved to that point. The process is repeated until there are no new assignments.

Clusters of words can be viewed as topics. The meaning of a topic is captured by the words in the cluster. For example, a topic containing the words “join”, “sports”, “team”, “joined”, “practice”, and “won” describes the topic of playing team sports. Thus, we can identify latent topics in a corpus of text by analyzing the clusters of words generated.

## SuicideWatch

In this section, we first present the data gathered and used in our analysis. Researchers interested in the code and the data are invited to contact the authors.

Reddit is a website which enables users to aggregate, rate and discuss news, entertainment, politics and many other topics. According to Alexa, it is the 8th most popular website in the world. It was estimated by the Pew research center that 6% of online adults use Reddit [26]. The site is organized into a collection of “subreddits”, each focused on a particular topic and administered by a collection of moderators.

The subreddit, r/SuicideWatch, is a forum in which online users are encouraged to post their thoughts regarding suicide. At the time of our data collection, it had over 58,000 subscribers. Sometimes users express a preoccupation with the thought of suicide. Other times users discuss immediate plans to take their own life. These posts often contain a description of their mental state including depression, reaction to stress, their feelings of being alone and having a low self-esteem.

While most online sources of data are notoriously noisy, this particular subreddit is remarkably clean. Given the serious nature of the subreddit, individuals are less likely to post harassing comments or off-topic remarks. When users post such comments, the moderators of the subreddit quickly remove them.

We collected all posts from its inception in 2008 to 2016. Each post is often commented on by other individuals. In this work, we focused on the original post as it most often represents the suicidal ideation of a user and comments often represent emotional support from other users.

We cleaned this data. First, we removed empty posts in which the content had been deleted. Second, we removed links, and replaced them with the word “link”. Third, we concatenated the text of the post to the title, as many users begin their post in the title and continue in the body of the post. Finally, we removed punctuation and other special characters. After cleaning this data, we had 131,728 posts with 27,978,246 words, of which 84,607 words were unique, posted by 63,252 unique users.

## Results

In this section, we evaluate the models built upon the r/SuicideWatch data. We begin by exploring individual words to subjectively assess whether or not the word representations are effectively capturing semantic information. We analyze the clusters to assess their ability to express latent topics in the data. We then evaluate the clusters by comparing them to the risk factors previously defined by domain experts.

### Experimental parameters

Once we obtained the data we began by creating vector representations using the Word2Vec from the gensim module for python [27]. Each post was processed using the window size of 5, common in the literature, which looks at the previous and next 5 words, along with the current word, looking at a total of 11 words at once.

Negative sampling was set to 20 from the default of 5 on the recommendation of the authors of the Word2Vec model, based on the size of our data [19, 20]. After extensive evaluation, we chose to represent words with a vector of 300 features using the skip-gram model and hierarchical softmax. This value seemed to provide rich semantic descriptions while minimizing computational overhead.

In order to preserve the meaning of phrases, we turned common phrases into single tokens, called n-grams. This allows phrases such as “new york” to be separate from “new” and “york” alone, which have very different meanings. This resulted in an increase in the size of vocabulary to 97,368 unique words and phrases. To avoid noise in the data, we set the minimum count for a word to be included as 10 occurrences. This removed noise in terms of misspelled words and unrecognized characters among other things. After filtering our vocabulary, we preserved 99.41% of all of the words in our data, which decreased our vocabulary to its final size of 28,663 unique words.

Next, we clustered the vector representations of words by using *k*-means clustering implemented by scikit-learn [28]. An important input to the algorithm is the selection of *k*. After extensive evaluation, we chose a value of 100 because it offered a sufficient number of clusters to capture the topics of the posts without being too large to manually evaluate. Regarding an error as the distance of each vector to its cluster center, we calculated the sum of the squared errors (SSE) for clusterings of size 5 through 400. The knee of the SSE curve was approximately 100 clusters.

To evaluate the clusters, we took the ten most common words from each cluster and attempted to assign the clusters to one of the twelve risk factors previously identified by experts in suicide ideation.

### Analysis of word representations

To visually inspect the effectiveness of our word representations, we first subjectively evaluated the representations of “heartbreak”, “pills”, and “knife”. Table 1 contains these query words along with the most similar words from the corpus. For example, the most similar token to “knife” is “kitchen knife”.

**Table 1:** Nearest words in vector space to test word

Heartbreak	Pills	Knife
Loneliness	Sleeping pills	Kitchen knife
Betrayal	Painkillers	Blade
Heartache	Tylenol	Razor
Sadness	Pain killers	Razor blade

In all three cases, the related words share meaningful semantic information. In the case of “knife”, the related words are synonyms. In the case of “pills”, the related words are specific types of pills such as “painkillers” or “tylenol”. In the case of “heartbreak”, the word representations appear to capture this emotional concept.

Now, after looking at semantic similarity, we attempted to see if our word vectors could be used for analogical reasoning in the same way they were used in [20]. Since words are represented as vectors, it is possible to add and subtract them from each other. We first consider the vector resulting from “[father] - [man] + [woman]”. We found that the vector representation most similar to the vector created by the preceding arithmetic is the vector representation of “mother”.

In addition to capturing general semantic meanings, our model also captured semantic information relevant to suicidal ideation. For example, when considering vector representations, we found that “[abusive] - [physical] + [words]” is most similar to “emotionally abusive”, and “[suicide] + [self]” is most similar to “killing myself”. This indicated to us that the word embeddings have captured semantic information relevant to the topic of suicidal ideation.

Finally, we observed that our model captured subtle distinctions between some similar words. This is demonstrated by the relation “[family] - [love] + [obligation]” being most similar to the word “relatives”. This example shows that even though “family” and “relatives” have many similar semantic components, our model is able to capture subtle distinctions in their meaning.

As the previous examples matched our intuitions, we believed that our model has effectively extracted significant semantic information from the corpus and is suitable for clustering to extract latent topics.

### Analysis of informal topics

To evaluate the clusters, we visually inspected the most common words in 100 clusters to see if they are related. For example one cluster contains the following terms: “since”, “past”, “suicidal”, “havent”, “times”, “attempt’, “attempted suicide”, “suicide attempt”, “almost killed”, and “failed attempt”. The words in this cluster discuss suicide attempts. Note that there are n-grams appearing in our clusters, indicating that the words constituting the phrases “attempted suicide”, “suicide attempt”, and “almost killed” were often used together in their respective phrases. These words when clustered together appear to capture the topic of past suicide attempts.

In another example, a cluster contains “physically”, “emotionally”, “bullied”, “treated”, “mentally”, “raped”, “ignored”, “rejected”, “abused”, and “abandoned”. These terms are mostly verbs describing some sort of abuse, both mental and physical. We observe that users often use these words when talking about physical abuse.

Finally, one cluster contains the terms “school”, “college”, “failed”, “class”, “university”, “grades”, “classes”, “failing”, “degree” and “major”. These terms are all used to describe education, especially higher education. While this cluster does not represent a risk factor for suicide, it does indicate that people often talk about college in the context of suicide, perhaps as a stressor that can lead to suicidal tendencies.

Some clusters capture concrete topics such as those containing “drugs” or “guns”. Still, others capture emotional topics such as those containing “anxiety” or “sadness”. Some clusters appear immediately relevant to the study of suicide such as those containing “cut” or “pain”, while others represent cohesive clusters but do not clearly represent topics related to suicide such as those containing “clothes” or “week”. While it is not possible to present all clusters here, a curated selection can be found in Table 2.

**Table 2:** Notable word clusters representing informal latent topics extracted from posts to r/SuicideWatch

Risk factor	Cluster 1	Cluster 2	Cluster 3	Cluster 4	Cluster 5
Bullying (8)	hit (5372)	against (3612)	mad (2645)	physically (3458)	started (21893)*
	turned (5016)	abuse (2218)	threatened (1054)	emotionally (2451)	high school (8846)
	broke (4541)	involved (1606)	fights (740)	bullied (2375)	hated (3034)*
	throw (2145)	behavior (923)	yelling (560)*	treated (2183)	dropped out (1830)*
	beat (2132)*	rape (767)*	bully (455)*	raped (1625)*	bullying (1005)*
Depressive feelings (10)	very (33446)	feel (125439)	no (109179)	happiness (5251)	into (32556)
	depressed (20645)	am (114263)	any (50721)	sense (5040)	fall (22057)
	become (9027)	feeling (26390)	real (12766)	sort (4433)	down (3249)
	angry (5752)	alone (25203)	future (10001)	lack (3606)	slowly (3151)
	extremely (4807)	sad (10933)	experience (5321)	desire (2629)	deep (2733)
Depressive symptoms (9)	these (16523)	day (36329)	room (8007)	pain (21604)	chest (2843)
	thoughts (13084)	days (14379)	crying (7023)	fear (6492)	stomach (1543)
	feelings (7829)	sleep (13630)	cried (2405)	constant (3805)	heavy (1047)
	suicidal thoughts (5999)	hours (10181)	tears (2356)*	sadness (2810)	panic attack (989)
	emotions (3258)	cry (7077)	screaming (1551)*	guilt (2749)	panic (983)
Drug Abuse (3)	pills (6482)	medication (5829)	drugs (5581)		
	bottle (2204)	meds (4811)	drunk (4560)		
	overdose (1451)	medicine (1537)	drinking (3660)		
	sleeping pills (1036)	antidepressants (1432)	alcohol (3006)		
	xanax (729)*	mg (1211)	weed (1423)*		
Family violence and discord (1)	father (9207)				
	kids (6564)				
	child (5125)				
	abusive (2063)				
	divorce (1508)*				
Gun ownership (0)					
Impulsivity (0)					
Prior suicide attempt (3)	since (32577)	hospital (8014)			
	past (17510)	admitted (1339)			
	suicidal (15001)	er (1121)			
	times (10523)*	hospitalized (1049)*			
	attempt (4238)*	committed (792)*			
Psychological disorders (6)	depression (26860)	therapist (6517)	problems (12848)	tried (30016)	results (836)
	anxiety (10311)	doctor (5205)	due (7429)	therapy (6619)	combination (513)
	diagnosed (3905)	psychiatrist (3084)	issues (5561)	doctors (2947)	ect (461) *‡*
	bipolar (1964)	treatment (1861)	stress (4894)	several (2791)	levels (455)
	social anxiety (1810)	mental health (1680)	emotional (2733)	medications (1264)	hormones (422)
Self-harm (2)	cut (7379)	body (7210)			
	cutting (3095)	heart (5707)			
	knife (2143)	blood (1618)*			
	wrists (1392)	burn (697)*			
	scars (1016)*	tear (562)*			
Suicide around individual (1)	family (41145) *†*				
	parents (30890)				
	kill herself (1327)*				
	kill himself (1064)*				
	committed suicide (971)*				
Suicide ideation (8)	thought (30326)	hanging (3298)	head (13150)	edge (2508)	plan (6527)
	suicide (25416)	hang (2736)	gun (4240)	near (2191)	easy (5018)
	thinking (20582)	rope (1836)	hand (3929)	jump (2177)	option (2907)
	mind (15745)	neck (1598)	pull (2406)	bridge (1764)	method (1576)
	killing myself (8223)	noose (811)	trigger (1577)	building (1684)	quick (1559)
Other important (23)	stupid (11266)	money (18076)	died (5854)	nice (7526)	girl (15934)
	such (11259)	pay (6742)	cancer (1974)	perfect (3430)	relationship (11081)
	man (6788)	debt (3360)	killed (1449)	beautiful (3388)	guy (10570)
	failure (6169)	cant afford (2526)	disease (995)	strong (3303)*	loved (8486)*
	selfish (5821)	rent (2136)	brain damage (216)*	smart (2577)*	broke up (4120)*
Accessory (26)	eat (4649)	internet (3720)	bought (1799)	years (41839)	phone (4797)
	food (3724)	music (3034)	clothes (1348)	year (22996)	online (4081)
	buy (2794)	watch (2706)	bag (687)	two (17720)	text (3848)
	water (1440)	computer (2481)	table (683)	months (13798)	contact (3480)
	pack (522)*	game (2426)	laptop (646)	week (13076)	message (2318)

Clusters are manually labeled according to risk factors proposed by experts. Gun Ownership and Impulsivity intentionally left blank, as none of the discovered clusters matched those risk factors. Additionally, other clusters were identified that held relevant semantic meaning, but didn’t fit into the risk factors. These were included under the label Accessory. Number next to risk factor indicates number of clusters with its label. Number next to word indicates frequency of occurrence in corpus. “*” incdicates that term is not among the top 5 common terms of cluster

### Comparison to risk factors

In our previous section, we showed how the clusters we found extract meaningful topics from the r/SuicideWatch data. In this section, we compare these informally extracted topics to risk factors proposed by domain experts. In this work, we draw from the risk factors used in Jashinsky et al., where Twitter data was analyzed according to risk factors identified by the National Institute of Mental Health and by Lewinsohn et al. [5, 10, 11]. The twelve risk factors can be seen in the left-hand column of Table 2.

While analyzing our clusters, we identified many topics that matched very closely with the proposed risk factors. For example, the notion of “Suicide Ideation” is captured by several clusters. For convenience, we have labeled the columns “Cluster 1” through “Cluster 5”, but there is no natural order to the clusters. On the row labeled “Suicide Ideation” we find that the first cluster expresses thoughts about committing suicide. The second, third, and fourth clusters discuss methods of committing suicide, and the fifth cluster portrays the user’s thoughts about planning suicide. Additionally, the risk factor “Self-Harm” also aligns well with our informal latent topics. Cluster one captures the notion of cutting oneself while cluster two focuses more clearly on damage to body parts such as “body”, “blood”, “burn” and many other words describing harm to one’s body. These topics both fit within the risk factor “Self-Harm”, showing agreement between our automatically generated topics and expert opinions. The informal topics captured by these clusters seem to embody the notion on suicidal ideation and suggest that our topics agree with the proposed risk factor.

We found that some clusters were not squarely matched with risk factors. For example, we assigned the cluster containing “mom”, “dad”, “kill herself” and “kill himself” to the risk factor “Suicide Around Individual”. This cluster also includes “friend”, “dog”, “gf”, “boyfriend” and a long list of other types of individuals in the user’s life. This informal latent topic seems to capture not only the occurrence of suicide but also examples of strong personal relationships, the loss of which could be particularly traumatic. Thus, this cluster relates to both of the risk factors “Family Violence and Discord” and “Suicide Around Individual”. In fact, there were conceptual overlaps in many clusters, especially those pertaining to depression, suicide ideation, psychological disorders, and self-harm.

In addition to finding more general topics, in some cases, the informal latent topics are more specific than the expertly derived risk factors. A good example of this is the risk factor “Drug Abuse” and the related informal topics. The first cluster represents the notion of “pills” and “sleeping pills”. The second cluster represents the notion of “medication” and “meds”. The third cluster represents the notion of “alcohol”, ‘drinking”, and recreational drugs such as “weed”. All of these clusters fit well under the heading of “Drug Abuse”, but vary significantly in their focus. The nuances in the discussions of drug abuse in online social media appear to result in topics capturing differing dimensions of this risk factor.

We also occasionally didn’t find clusters associated with risk factors. Despite “Gun Ownership” previously being identified as an important risk factor [11], we were unable to find a cluster which explicitly represented the idea of owning a gun. We did find the word, “gun”, in our clusters as well as many related words such as “shoot”. However, these words are clustered with terms related to suicidal thoughts rather than ownership. This example highlights one of the main differences between the expertly derived risk factors and the informal latent topics extracted from social media. While it may be true that those who have access to a gun are at greater risk to commit suicide, it does not appear that those who express suicide ideation online reference their ownership of a gun with as much clarity as they discuss other topics.

Some clusters were particularly difficult to classify. The clusters corresponding to “Depressive Feelings” and “Depressive Symptoms” were difficult to differentiate. The Anxiety and Depression Association of America lists symptoms of depression as, among other things, irritability, insomnia, fatigue, difficulty making decisions, persistent physical symptoms, and feelings of hopelessness, worthlessness and helplessness.

Many users discuss their depression, not as a dichotomy between feelings and symptoms, but instead use the words more casually. When assigning clusters to risk factors, we attempt to make a distinction between feelings and symptoms. Symptoms can be identified as physical ailments or development of disorders and conditions such as anxiety, sadness, and stress. Feelings tend to be a more nuanced description of one’s self and experience.

One cluster contains the words “no”, “any”, “real”, “future”, “real”, “experience”, “motivation”, “social”, “dreams”, “purpose”, and “plans”. We classified this cluster as depressive feelings because the words seem to indicate a lack of purpose and a sense of uselessness. On the other hand, the cluster which contains “these”, “thoughts”, “feelings”, “suicidal thoughts”, “emotions’ and “panic attacks” is more focused on symptoms of depression that one may face.

Regardless of whether or not a word is labeled as a symptom or as a feeling, our informal latent topics often capture very specific depressive language. One cluster contains “depressed”, “angry” and “upset” capturing common emotional keywords. Another cluster contains “chest”, “stomach” and “heavy” describing the physical reaction to stress. A third contains “into”, “fall” and “down” using the familiar imagery of downward movement when describing depression. Indeed, we found a total of nineteen clusters relevant to depressive feelings and symptoms, a few of which are presented in Table 2. The diversity and specificity of our informal latent topics seem to capture subtle differences in how users discuss suicide in online posts.

We found other clusters which we could not label according to the twelve suicide risk factors, and which we accordingly labeled “Other Important”. These clusters were identified as possible contributors to suicide ideation, and contain information which we determined may be valuable to identify and assess suicide risk in social media posts. For example, one cluster includes “stupid”, “failure” and “selfish”. Authors of the posts often use these words to describe their self-image. Another cluster includes “died”, “cancer”, and “disease” presenting the occurrence of a serious medical condition in the user’s life.

In all, we identified 22 important clusters that did not fit well into the 12 previously proposed risk factors, many representing stressors that might lead to suicidal ideation. Other topics include poor performance in school, trouble with money, and disgust with one’s physical appearance.

The complexity of natural language often made it challenging to categorize the informal topics. For example, positive words are sometimes used to express negative feelings. A cluster containing mainly positive tokens such as “nice”, “beautiful”, “perfect”, “strong”, and “smart” may be referencing legitimately positive characteristics. On the other hand, a user might be posting about how good the life of other people seems to be while their life is lacking. Examples of these sentiments from posts are, “My family acts so perfect and seems so perfect from the outside” and “Why is everyone else so beautiful?”.

Finally, many of the latent topics did not seem immediately relevant to suicidal ideation but were often present in the online posts. Five of these clusters are shown in the last row of Table 2. For example, one cluster represents the notion of food while another represents clothes.

## Discussion

To evaluate our models, we first subjectively evaluated the latent topics represented by clusters of words. We then compared these topics to risk factors generated by domain experts. Our in-depth analysis revealed several key findings.

First, we found that the topics discovered by our analysis had a large scope. Topics ranged from crying to clothing to the calendar. This illustrates that our model was able to identify different latent topics within the corpus and separate them into meaningful clusters. It also shows that there are topics that people discuss which are not directly related to suicide, as not every word is on the topic of suicide.

When comparing our automatically generated topics to previously identified risk factors, we found that there were some differences in the focus of the topics compared to that of the risk factors. In the case of “Drug Abuse”, people tended to discuss recreational drugs, specifically alcohol, separately from medications and pills. This difference in focus shows how the public view of these two topics may fit under the umbrella term provided by experts, but differ enough to be separate topics. On the other hand, in the case of “Family Violence and Discord” and “Suicide Around Individual”, the topics generated by our model seemed to indicate a broader topic, rather than topics as specific as these risk factors.

A result of collecting data from public users with presumably no professional medical experience is the difference in precision of language between users and medical professionals. An indicator of this difference is in discussing depression. While professionals made a difference between “Depressive Feelings” and “Depressive Symptoms”, the topics identified from users’ posts overlapped these ideas. This may be partly due to the fact that depressive feelings are a symptom of depression, but also to a lack in precise use of language to describe specific experiences and symptoms.

Our contribution to this field is the discovery of latent topics within textual data known to contain suicidal ideation. A common method for identifying suicidal ideation in social media is to use a filter designed by medical professionals to extract data. Such a technique may impose a structure on the data by medical professionals that does not reflect the actual language used by those experiencing suicidal ideation. Our method uses topic modeling to uncover informal, latent topics directly from social media posts, which captures the ideas deemed important by those who are sharing their experiences with an online community. This information will inform the medical community which informal topics are important to monitor in informal contexts, such as social media, to effectively identify suicidal ideation.

## Conclusion

In this work, we automatically extracted informal latent topics from online social media expressing suicidal ideations. We first subjectively evaluated the latent topics and then exhaustively compared them to risk factors proposed by domain experts. In general, we found that our informal topics are similar to the expert’s risk factors; however, our topics differ in several important ways. Our topics can be more specific or more general. Some of our topics express meaningful ideas not contained in the risk factors and some risk factors do not have complimentary latent topics. In short, our analysis of the latent topics extracted from social media containing suicidal ideations suggests that users of these systems express ideas that are complementary to the topics defined by experts but differ in their scope, focus, and precision of their language.

This effort opens up many possibilities for future work. First, we will build models leveraging the informal topics to predict the urgency of the posts. Second, we plan to compare these results to other topic modeling algorithms such as latent Dirichlet analysis and latent semantic analysis. Finally, we will extend our analysis to other mental health issues such as post-traumatic stress disorder and depression.

## Abbreviations

CBOW: Continuous bag of words
LIWC: Linguistic inquiry and word count
SSE: Sum of squared errors

## References

[1] Nock MK, Borges G, Bromet EJ, Alonso J, Angermeyer M, Beautrais A, Bruffaerts R, Chiu WT, De Girolamo G, Gluzman S (2008). Cross-national prevalence and risk factors for suicidal ideation, plans and attempts. Br J Psychiatr.

[2] Association AP (2013). Diagnostic and Statistical Manual of Mental Disorders (DSM-5)..

[3] Hawton K, van Heeringen K (2009). Suicide. The Lancet.

[4] Coppersmith G, Dredze M, Harman C (2014). Quantifying mental health signals in twitter. Proceedings of the Workshop on Computational Linguistics and Clinical Psychology: From Linguistic Signal to Clinical Reality.

[5] Jashinsky J, Burton SH, Hanson C, Argyle T (2013). Tracking suicide risk factors through twitter in the US. Crisis J Crisis Interv Suicide Prev.

[6] Pennebaker JW, Francis ME, Booth RJ (2001). Linguistic inquiry and word count: Liwc 2001. Mahway: Lawrence Erlbaum Associates.

[7] De Choudhury M, Gamon M, Counts S, Horvitz E (2013). Predicting depression via social media. Proceedings of the Seventh International AAAI Conference on Weblogs and Social Media.

[8] Kumar M, Dredze M, Coppersmith G, De Choudhury M (2015). Detecting changes in suicide content manifested in social media following celebrity suicides. Proceedings of the 26th ACM Conference on Hypertext & Social Media.

[9] O’Dea B, Wan S, Batterham P, Calear A, Paris C, Christensen H (2015). Detecting suicidality on twitter. Internet Interv.

[10] Lewinsohn PM, Rohde P, Seeley JR (1994). Psychosocial risk factors for future adolescent suicide attempts. J Consult Clin Psychol.

[11] Suicide in the U.S. (2012). Statistics and Prevention.

[12] Bamidis P, Coppersmith G, Spitzberg B, LItman L, Tsou M-H, Konstantinidis S, Braithwaite SR, Giraud-Carrier C, West J, Barnes MD, Hanson CL. Validating machine learning algorithms for twitter data against established measures of suicidality. J Med Internet Res Ment Health. 2016; 3(2). https://doi.org/10.2196/mental.4822. 10.2196/mental.4822 PMC4886102 27185366

[13] De Choudhury M, Kiciman E, Dredze M, Coppersmith G, Kumar M (2016). Discovering shifts to suicidal ideation from mental health content in social media. Proceedings of the 2016 CHI Conference on Human Factors in Computing Systems. CHI ’16.

[14] Gkotsis G, Oellrich A, Hubbard T, Dobson R, Liakata M, Velupillai S, Dutta R. The language of mental health problems in social media. In: The Third Computational Linguistics and Clinical Psychology Workshop (CLPsych): 2016. p. 63–73.

[15] Coppersmith G, Ngo K, Leary R, Wood A (2016). Exploratory analysis of social media prior to a suicide attempt. Proceedings of the Third Workshop on Computational Linguistics and Clinical Psychology.

[16] Csurka G, Dance CR, Fan L, Willamowski J, Bray C (2004). Visual categorization with bags of keypoints. In Workshop on Statistical Learning in Computer Vision.

[17] Hofmann T. Probabilistic latent semantic analysis. In: Proceedings of the Fifteenth Conference on Uncertainty in Artificial Intelligence. Morgan Kaufmann Publishers Inc.: 1999. p. 289–96.

[18] Blei DM, Ng AY, Jordan MI (2003). Latent dirichlet allocation. J Mach Learn Res.

[19] Mikolov T, Chen K, Corrado G, Dean J. Efficient estimation of word representations in vector space. 2013. arXiv preprint arXiv:1301.3781.

[20] Mikolov T, Sutskever I, Chen K, Corrado GS, Dean J. Distributed representations of words and phrases and their compositionality. Lake Tahoe, Nevada: Advances in Neural Information Processing Systems: 2013. p. 3111–9.

[21] Hong L, Davison BD (2010). Empirical study of topic modeling in twitter. Proceedings of the First Workshop on Social Media Analytics.

[22] Zanotti G, Horvath M, Barbosa LN, Immedisetty VTKG, Gemmell J (2016). Infusing collaborative recommenders with distributed representations. Proceedings of the 1st Workshop on Deep Learning for Recommender Systems.

[23] Wolf L, Hanani Y, Bar K, Dershowitz N (2014). Joint word2vec networks for bilingual semantic representations. Int J Comput Linguistics Appl.

[24] Salton G, Wong A, Yang CS (1975). A vector space model for automatic indexing. Commun ACM.

[25] Hartigan JA, Wong MA (1979). Algorithm as 136: A k-means clustering algorithm. J R Stat Soc Ser C (Appl Stat).

[26] Duggan M, Smith A (2013). 6% of online adults are reddit users. Pew Internet Am Life Proj.

[27] Řehůřek R, Sojka P (2010). Software Framework for Topic Modelling with Large Corpora. Proceedings of the LREC 2010 Workshop on New Challenges for NLP Frameworks.

[28] Buitinck L, Louppe G, Blondel M, Pedregosa F, Mueller A, Grisel O, Niculae V, Prettenhofer P, Gramfort A, Grobler J, Layton R, VanderPlas J, Joly A, Holt B, Varoquaux G. API design for machine learning software: experiences from the scikit-learn project. Prague: ECML PKDD Workshop: Languages for Data Mining and Machine Learning: 2013. p. 108–22.

